# West Nile Virus-Induced Expression of Senescent Gene *Lgals3bp* Regulates Microglial Phenotype within Cerebral Cortex

**DOI:** 10.3390/biom14070808

**Published:** 2024-07-08

**Authors:** Artem Arutyunov, Violeta Durán-Laforet, Shenjian Ai, Loris Ferrari, Robert Murphy, Dorothy P. Schafer, Robyn S. Klein

**Affiliations:** 1 Center for Neuroimmunology & Neuroinfectious Diseases, St. Louis, MO 63110, USA; artem@wustl.edu (A.A.); shenjian.ai@wustl.edu (S.A.); 2 Department of Medicine, Washington University School of Medicine, St. Louis, MO 63110, USA; 3 Department of Neurobiology, Brudnick Neuropsychiatric Research Institute, University of Massachusetts Chan Medical School, Worcester, MA 01655, USA; violeta.duranlaforet@umassmed.edu (V.D.-L.); loris.ferrari@umassmed.edu (L.F.); robert.murphy@umassmed.edu (R.M.); dorothydori.schafer@umassmed.edu (D.P.S.); 4 Department of Microbiology & Immunology, Western Institute of Neuroscience, Schulich School of Medicine & Dentistry, University of Western Ontario, 100 Perth Dr, London, ON N6A 5K8, Canada

**Keywords:** microglia, neuroinfectious disease, flavivirus encephalitis, neurodegeneration, aging, microglia transcriptomics, CD8 T cell, Lgals3bp, Lgals3, senescence, West Nile virus

## Abstract

Microglia, the resident macrophages of the central nervous system, exhibit altered gene expression in response to various neurological conditions. This study investigates the relationship between West Nile Virus infection and microglial senescence, focusing on the role of LGALS3BP, a protein implicated in both antiviral responses and aging. Using spatial transcriptomics, RNA sequencing and flow cytometry, we characterized changes in microglial gene signatures in adult and aged mice following recovery from WNV encephalitis. Additionally, we analyzed *Lgals3bp* expression and generated *Lgals3bp*-deficient mice to assess the impact on neuroinflammation and microglial phenotypes. Our results show that WNV-activated microglia share transcriptional signatures with aged microglia, including upregulation of genes involved in interferon response and inflammation. *Lgals3bp* was broadly expressed in the CNS and robustly upregulated during WNV infection and aging. *Lgals3bp*-deficient mice exhibited reduced neuroinflammation, increased homeostatic microglial numbers, and altered T cell populations without differences in virologic control or survival. These data indicate that LGALS3BP has a role in regulating neuroinflammation and microglial activation and suggest that targeting LGALS3BP might provide a potential route for mitigating neuroinflammation-related cognitive decline in aging and post-viral infections.

## 1. Introduction

Microglia, the yolk sac progenitor-derived resident macrophages of the central nervous system, exhibit altered transcriptomic signatures in a broad spectrum of neurological conditions, ranging from viral infections to aging and neurodegenerative diseases. Changes in gene expression of microglia indicate a shift in their functions from homeostatic maintenance of neural networks to induction of inflammation or repair, depending on the disease. Although aging is a normal process, studies of senescent markers within microglia reveal an overlap between genes characteristic of aged microglia and those involved in interferon (IFN) signaling pathways [1], specifically *Ifitm3*, *Ifi204*, *Cxcl16*, *Gas6* and others. IFN responses are essential for virologic control [2,3,4], and infections with neurotropic viruses have been linked to neurodegeneration [5,6]. However, the links between virus-mediated inflammation and microglial senescence are poorly understood.

West Nile virus (WNV) is a mosquito-borne arbovirus belonging to the *Flaviviridae* family of viruses [7], which also includes other human disease-causing viruses such as Japanese Encephalitis, Zika (ZIKV), Dengue and Yellow Fever viruses. WNV infection in humans can progress to West Nile Encephalitis, which presents with severe neurological symptoms including meningitis, encephalitis, and paralysis, progressing, in some cases, to coma, with an overall mortality of 10% [8]. Long-term cognitive and memory impairments occur in the majority of survivors and resolve in months or persist and worsen indefinitely [9]. Using WNV-NS5-E218A, a strain of WNV containing a single point mutation in the gene encoding 2′-O-methyltransferase that enables clearance by Type 1 interferon responses [10], our lab has previously published data characterizing microglial transcriptomic changes during recovery from WNV. These changes include, but are not limited to, increased expression of genes involved in interferon response, including *Ifitm3*, *Ifi204*, *Itm2b*, *Cxcl16*, and *Lgals3bp.* Importantly, we found that almost all microglia become activated in the forebrain of WNV-recovered mice, suggesting loss of their homeostatic functions [11,12]. In addition, studies of T cell function during WNV recovery demonstrate that resident memory CD8 T cells maintain microglia activation via Type 2 interferon signaling [12,13].

LGALS3BP, a heavily glycosylated protein, is a known binding partner for Galectin 3 (LGALS3), which is expressed by a wide array of cells and present in most biological fluids, including serum, cerebrospinal fluid, saliva, and others. Early studies of LGALS3BP focused on its roles during HIV infection, where it was shown to be a serum marker for progression to AIDS. LGALS3BP expression is also elevated in carcinogenesis, including lung, breast [14], glioblastoma multiforme (GBM) [15] and others. Exact molecular mechanisms of LGALS3BP action, as well as the downstream effects of its interaction with LGALS3, are unknown, but there is evidence for Lgals3bp being involved, paradoxically, in both IFN- [16] and TGFβ [17]-mediated immune response pathways. *Lgals3bp* deficiency has previously been shown to promote increased severity and mortality from Influenza type A, and higher viral loads of Zika, Herpes Simplex and Vesicular Stomatitis Viruses in mice [16]. More recently, increased LGALS3BP levels in cerebrospinal fluid were shown to be significantly correlated with advanced age in humans [18]. In addition, patients with point mutations in LGALS3BP have altered cortical gyrification and thickness, sulcal depth and surface area, suggesting potential roles for LGALS3BP in the regulation of neural progenitor positioning [19].

Given these roles for LGALS3BP at the intersection of antiviral response and aging, we hypothesized that neuroinflammation caused by WNV infection might drive expression of senescence-related genes within microglia that differentially regulate neuroinflammation. Here, we used spatial imaging and RNA sequencing to characterize virus-mediated alterations in microglial gene signatures in both adult and aged mice after recovery from WNV encephalitis. We found that while activated microglia in the WNV-recovered forebrain share transcriptional signatures with aged/senescent microglia, many of these gene alterations may be infection- and aging-dependent. Among these genes, *Lgals3bp*, which we found to be broadly expressed in the CNS by microglia, astrocytes, and neurons, regulates microglial phenotype and numbers of infiltrated T cells in the forebrain of WNV-recovered animals.

## 2. Materials and Methods

### 2.1. Viruses and Infection Procedures

The WNV-NS5-E218A strain used in this study contains a single point mutation in the gene encoding 2′-O-methyltransferase and was constructed previously [20,21] from WNV 3356 strain and passaged once in Vero cells [10]. Mice were deeply anesthetized with a cocktail of ketamine/xylazine/acepromazine and intracranially administered 10^4^ plaque-forming units (p.f.u.) of WNV-NS5-E218A. Virus was diluted in 10 μL of 0.5% fetal bovine serum (FBS) in Hank’s balanced salt solution (HBBS, ThermoFisher, Waltham, MA, USA). Virus or diluent (mock) was injected into the third ventricle of the brain with a guided 29-gauge needle, as previously described.

### 2.2. Animals

All mouse experiments adhered to the guidelines approved by the Washington University in St. Louis Institutional Animal Care and Use Committee under IACUC protocol number 21-0019. All experiments were approved by the institutional biological and chemical safety committee at Washington University in St. Louis. All mice used in these experiments were male or female C57BL6/J inbred mice obtained commercially (The Jackson Laboratory) at specified ages and housed in pathogen-free facilities at the Washington University School of Medicine.

### 2.3. Viral Burden Measurements

Mice were infected with WNV and euthanized at specific days post-infection, as indicated. For tissue collection, mice were deeply anesthetized with ketamine/xylazine/acepromazine and perfused with 20 mL of sterile, ice-cold Dulbecco’s phosphate-buffered saline (dPBS; ThermoFisher, Waltham, MA, USA). Tissues were snap frozen on dry ice, weighed, and then homogenized in 500 μL sterile dPBS. Viral titers were quantified by standard plaque assay with BHK21 cells, as described [22].

### 2.4. Leukocyte Isolation and Flow Cytometry

For flow cytometry experiments, leukocytes were isolated as described previously [23]. Mice were deeply anesthetized with ketamine/xylazine/acepromazine cocktail, then transcardially perfused with 20 mL dPBS, and the brain was removed. Brain tissue was minced and digested in HBSS containing 0.05% collagenase D (Sigma, Burlington, MA, USA), 0.1 μg/mL TLCK trypsin inhibitor (Sigma, Burlington, MA, USA), 10 μg/mL DNase I (Sigma, Burlington, MA, USA), and 10 mM Hepes pH 7.4 (Gibco, Grand Island, NY, USA) for 1 h at 37 °C with shaking. Tissue was then pushed through a 70 μm strainer and centrifuged at 500× *g* for 10 min. Brain cell pellets were resuspended in 37% isotonic Percoll (Cytiva, Marlborough, MA, USA) and centrifuged at 1200× *g* for 30 min to remove myelin debris, and the pellet was resuspended in dPBS. For ex vivo restimulation, isolated cells were then treated with 1 μg/mL ionomycin and 5 ng/mL phorbol myristate acetate to stimulate cytokine expression and 5 μg/mL Brefeldin A to block cytokine exocytosis for 4 h at 37 °C, 5% CO_2_. Prior to immunostaining, all cells were blocked with 1:50 TruStain FcX anti-mouse CD16/32 (Clone 93, Cat 101320 Biolegend, San Diego, CA, USA) for 5 min. Cells were stained with Live/Dead Fixable Blue at 1:1000 following dissolution in 40 μL DMSO (Invitrogen L34962, Waltham, MA, USA) and extracellular antibodies, as indicated, for 15 min at 22 °C, then washed twice with dPBS, fixed and permeabilized with Foxp3/Transcription Factor Staining Kit (eBioscience, 00-5523-00, San Diego, CA, USA). Intracellular markers were detected with antibodies as indicated for 15 min, then cells were washed twice with permeabilization buffer and twice with dPBS, then fixed with 2% paraformaldehyde (PFA). Data were collected with a BD LSR Fortessa X-20 flow cytometer and analyzed with FlowJo software v10.5.2.

### 2.5. Flow Cytometry Antibodies and Tetramers

All antibodies used at 1:200: CD11b (Clone M1/70, Cat 10137, Biolegend, San Diego, CA, USA), CD45 (Clone 30-F11, Cat 56-0451-82, eBioscience, San Diego, CA, USA), MHCII (Clone M5/114.15.2, Cat 107626, Biolegend, San Diego, CA, USA), CD80 (Clone 16-10A1, Cat 104706, Biolegend, San Diego, CA, USA), CD103 (Clone 2E7, Cat 48-1031-80, eBioscience, San Diego, CA, USA), CD8a (Clone 53-6.7, Cat 100712, Biolegend, San Diego, CA, USA), CD4 (Clone RM4-5, Cat 550954, BD Biosciences, Franklin Lakes, NJ, USA), CD44 (Clone IM7, Cat 103011, Biolegend, San Diego, CA, USA), P2RY12 (Clone S16007D, Cat 848006, Biolegend, San Diego, CA, USA), IFNγ (Clone XMG1.2, Cat 505826 Biolegend, San Diego, CA, USA), CD68 (Clone FA-11, Cat 137017 Biolegend, San Diego, CA, USA). WNV-specific CD8^+^ T cells were identified with fluorescent-labeled immunodominant Db-restricted NS4B peptide.

### 2.6. RNA Isolation and qRT-PCR

Following transcardiac perfusion, tissues were collected in 500 μL TRI Reagent (Cat AM9738, ThermoFisher, Waltham, MA, USA) and homogenized with zirconia beads using a MagNA Lyser instrument (Roche Life Science, Basel, Switzerland). RNA was subsequently isolated using Direct-zol RNA miniprep kit (Cat R2052, Zymo Research, Irvine, CA, USA) according to manufacturer’s instructions. After RNA concentration was measured using Nanodrop 2000 instrument (ThermoFisher, Waltham, MA, USA), iScript Reverse Transcription Supermix kit (Cat 1708841, Bio-Rad, Hercules, CA, USA) was used to generate cDNA. qPCR was carried out using SsoAdvanced Universal SYBR Green reagents (Cat 1725271, Bio-Rad, Hercules, CA, USA) with target-specific primers on a Bio-Rad CFX384 instrument according to manufacturer recommendations.

### 2.7. RNA Sequencing

For bulk RNA-sequencing, leukocytes were isolated as described above, with an additional step of CD11b^+^ cell enrichment by using CD11b magnetic microbeads (Cat 130-093-636, Myltenyi Biotec, Bergisch Gladbach, Germany). RNA was subsequently isolated from these cells using QIAgen RNeasy Micro kit (Cat 74134, QIAGEN, Hilden, Germany). Samples were prepared according to the library kit manufacturer’s protocol, indexed, pooled, and sequenced on an Illumina NovaSeq 6000. Basecalls and demultiplexing were performed with Illumina’s bcl2fastq2 v2.20 software. RNA-seq reads were then aligned and quantitated to the Ensembl release 101 primary assembly with an Illumina DRAGEN Bio-IT on-premise server running version 3.9.3-8 software. Gene counts were then analyzed in Partek Flow software v11.0.24.0604 suite using standard pipeline for bulk RNA-sequencing analysis to generate a list of differentially expressed genes (DEGs).

### 2.8. Multiplexed Error-Robust In Situ Hybridization (MERFISH): Sample Preparation and Imaging

Mice were deeply anesthetized using a ketamine/xylazine/acepromazine cocktail and then perfused transcardially with ice-cold HBSS. Extracted brains were embedded in OCT, flash frozen, and stored at −80 °C. The preparation of fresh frozen tissue samples followed Vizgen’s protocol (Vizgen, Cambridge, MA, USA). Brains were sectioned and mounted on Vizgen’s bead-coated functionalized coverslip. After adhering to the coverslip, samples were fixed in 4% paraformaldehyde (PFA) in 1× PBS for 15 min at room temperature, followed by three washes in 1× PBS for 5 min each. The samples were then incubated overnight in 70% ethanol to permeabilize the tissue. Next, the samples were incubated for 30 min in Formamide Wash Buffer (30% deionized formamide in 2× SSC), after which the gene library mix was added for the hybridization step, lasting 48 h at 37 °C. Samples were then washed twice with Formamide Wash Buffer for 30 min each at 47 °C. The tissue was then embedded in a gel embedding solution (containing 0.5% ammonium persulfate, 0.05% TEMED, 4% 19:1 acrylamide/bis-acrylamide solution, 0.3M NaCl, and 0.06M Tris pH 8) and incubated overnight with tissue clearing solution (2× SSC, 2% SDS, 0.5% Triton X-100, and proteinase K 1:100) at 37 °C. Finally, the tissue was washed, incubated with DAPI and polyT solution for 15 min at room temperature, and washed with formamide wash buffer for 10 min at room temperature. After sample preparation, the MERSCOPE 500 gene imaging kit was activated by adding 250 μL of Imaging Buffer Activator and 100 μL of RNAse Inhibitor. Fifteen milliliters of mineral oil was layered on top of the imaging buffer through the activation port, the instrument was primed, and the imaging chamber was assembled according to the MERSCOPE user guide. A 10x low-resolution mosaic of the sample was then acquired, the imaging area was selected, and the sample was imaged.

### 2.9. MERFISH: Post-Imaging Data Processing and Analysis

After image acquisition, the data were decoded utilizing Vizgen’s analysis pipeline integrated within the MERSCOPE system. Subsequently, the postprocessing tool from Vizgen was employed to achieve cell segmentation based on DAPI staining, utilizing the CellPose algorithm. Segmentation was conducted on the middle Z plane (3rd out of 7), with cell borders propagated to the adjacent Z planes above and below. The processed MERFISH data were analyzed in RStudio using Seurat 4.1.3, R 4.2.2, and custom scripts. Cells were filtered from the dataset to exclude those with a volume of less than 100 µm^3^, fewer than 10 unique transcripts, and fewer than 40 transcript counts. Gene expression per cell was normalized relative to each cell’s volume and the mean RNA per sample. For cell clustering, we employed a modified Seurat pipeline, performing principal component analysis (PCA) on 400 genes as the variable features, followed by dimensionality analysis with a resolution of 1.6 and 22 dimensions. Dimensionality reduction was achieved using Uniform Manifold Approximation and Projection (UMAP). Cell clusters were manually annotated based on the expression of commonly used cell-type-specific gene markers and their spatial distribution.

### 2.10. Gene Module Score Calculation

The module score for the senescent cells was calculated with the AddModuleScore Seurat function with 5 control features from the same bin per analyzed feature. Cells were considered senescent if the score was higher than 3 times the standard deviation + mean of the 16-week mock group.

### 2.11. Generation of Lgals3bp^−/−^ C57BL6/J Mice

All animal experiments were approved by institutional IACUC protocols. C57BL/6J mice at 3–4 weeks of age (JAX Laboratories) were superovulated by intraperitoneal injection of 5 IU pregnant mare serum gonadotropin, followed 47 h later by intraperitoneal injection of 5 IU human chorionic gonadotropin (Millipore-Sigma, Burlington, MA, USA). Mouse zygotes were obtained by mating C57BL/6J stud males with superovulated C57BL/6J females at a 1:1 ratio. One-cell fertilized embryos were electroporated with an RNP containing 12 μg of Cas9 protein, 2 μg of each gRNA, and 100 pmol (~5 μg) of each ssODN complex. Electroporations and mouse transgenesis experiments were performed as described previously [24,25,26].

### 2.12. Lgals3bp^−/−^ Mouse Genotyping

The target region was PCR-amplified by tailed primers (gene-specific primer sequences below) appended with 5′-CACTCTTTCCCTACACGACGCTCTTCCGATCT-3′ for forward and 5′-GTGACTGGAGTTCAGACGTGTGCTCTTCCGATCT-3′ for reverse to genomic-specific primer sequences (PCR1), which allowed unique indexes and Illumina P5/P7 adapter sequences to be added in a second round PCR. PCR amplifications were performed with SuperFi II 2x Platinum Green master mix (ThermoFisher, Waltham, MA, USA) according to the manufacturer protocol. Indexing of the PCR1 product was performed by using 0.1× volume from PCR1 with indexing primers (0.1 μM final concentration for each) and melting at 98 °C for 2 min, followed by five cycles of 98 °C for 15 s, 60 °C for 15 s, and 72 °C for 40 s. We generated 2 × 250 reads with the Illumina MiSeq platform at the Center for Genome Sciences and Systems Biology (Washington University). The extracted FASTQ files were analyzed by using a Python-based alignment script.

gRNA:

XCD628c.m.Lgals3bp.sp30: CAGGGCCCGGCAGACGACGT

Primers:

XCD628c.m.Lgals3bp.F: TGGGGAGTGAACATCAAACCA

XCD628c.m.Lgals3bp.R: CCAGCATGATCGGTCCCTTT

### 2.13. Statistical Analysis

Statistical analyses were performed using Prism 10.0 (GraphPad Software). All data were analyzed using an unpaired t test or two-way ANOVA with Sidak’s post-test to correct for multiple comparisons, as indicated in corresponding figure legends. A *p* value < 0.05 was considered significant.

## 3. Results

### 3.1. Microglia Activated by WNV Share Transcriptional Signatures with Aged/Senescent Microglia

**Figure 1:**
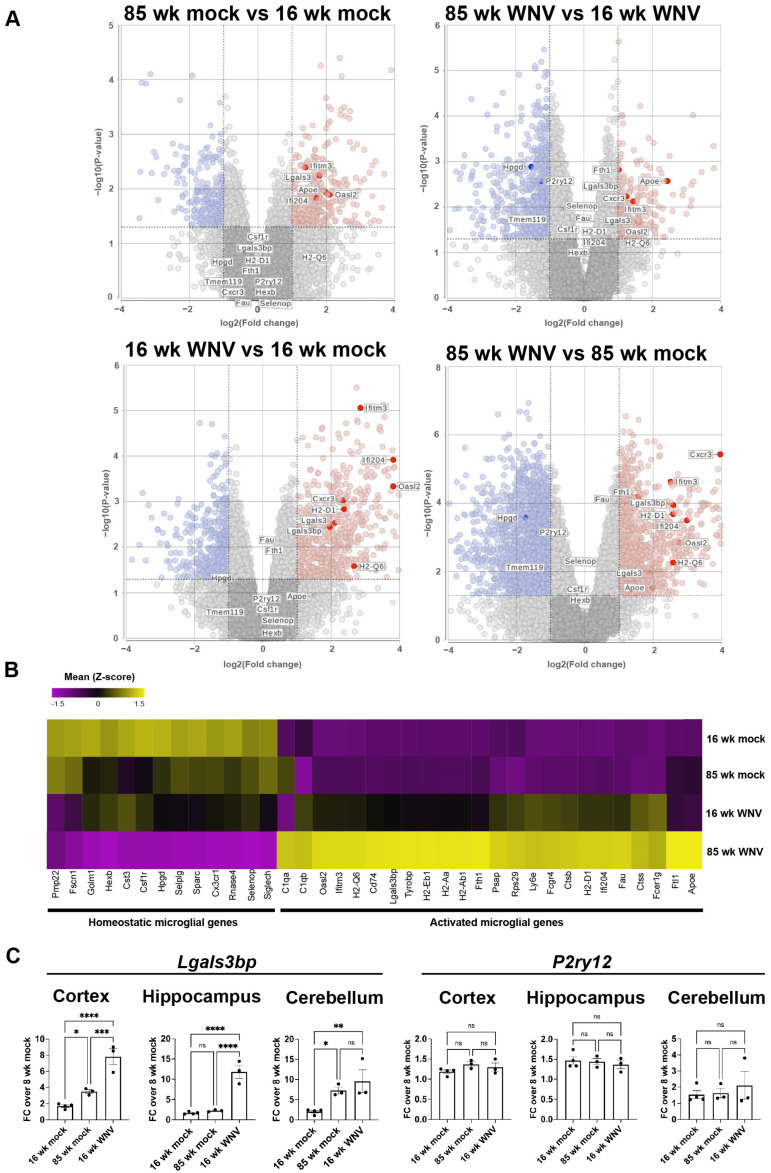
Microglia activated by WNV share transcriptional signatures with aged microglia. (**A**) Volcano plots showing pairwise comparisons of microglial gene expression in CD11b^+^ cells from brains of mock or WNV-infected 16- and 85-week-old mice analyzed by bulk RNA sequencing at 30 DPI. Genes in red and blue are up- or downregulated, respectively. (**B**) Data from the same experiment plotted as a heatmap, N = 3 for each sample type. (**C**) qRT-PCR validation of *Lgals3bp* and *P2ry12* expression in brain lysates obtained from 16- or 85-week-old mice. Statistical significance was calculated using one-way ANOVA with pairwise comparisons and Tukey’s correction test, * *p* < 0.05, ** *p* < 0.01, *** *p* < 0.0005, **** *p* < 0.0001, ns = not significant.

We previously published a murine model of recovery after intracranial infection with WNV-NS5-E218A, which has a single amino acid mutation in a 2′O methyltransferase that normally prevents viral RNA recognition by interferon-induced protein with tetratricopeptide repeats (IFIT1) [10]. Intracranial inoculation of WNV-NS5-E218A (WNV) leads to uniform infection across CNS regions, which is cleared by 15 days post-infection (dpi), with a survival rate and memory impairments similar to human survival after neuroinvasive WNV infection, and persistent microglial activation [11,27]. While the overlap between inflammation and aging has been explored, it is unknown whether a viral stimulus can trigger senescent phenotypes in the CNS, particularly in microglia. To address this, we compared previously published genes characteristic of activated or homeostatic microglia in our WNV recovery model [12] to published datasets of transcriptomic signatures of young (6 months) and aged (23 months) microglia [28]. This comparison revealed broad similarities in gene expression between WNV-activated and aged microglia (Table 1). Of note, expression of genes defining homeostatic microglia remain mostly unchanged during aging, while many of these genes are down-regulated during viral infection [29,30]. To build on these initial findings, we performed a bulk RNA-sequencing analysis of CD11b^+^ cells isolated from the forebrains of WNV-infected 16- or 85-week-old animals at 30 DPI. At this time-point, infiltrating monocytes are no longer present, leaving only homeostatic and activated microglia within the myeloid niche [11,12]. This analysis confirmed what we have observed in the published dataset of aged microglia: homeostatic genes *P2ry12*, *Selenop* and *Hexb* were unchanged between uninfected 85- and 16-week-old animals, while a number of genes characteristic of microglial activation, such as microglial *Oasl2*, *Ifitm3* and *Ifi204*, were increased in aged microglia (Figure 1A). WNV infection, in accordance with our previous data, resulted in robust upregulation of activated microglial genes in both 16- and 85-week-old animals (Figure 1B). Most of these genes were similar in expression between infected 16- and 85-week-old animals, however some genes, including *Ifitm3*, *Apoe* and *Fth1*, were expressed at significantly higher levels in aged infected microglia versus those derived from younger adult animals. Comparing uninfected 16- and 85-week-old mice revealed that aging also resulted in an increase in most genes characteristic of activated microglia, albeit to a lesser extent compared to WNV infection (Figure 1B). We validated these results in separate cohorts via qRT-PCR for representative homeostatic and activated genes, *P2ry12* and *Lgals3bp*, respectively (Figure 1B). *Lgals3bp* mRNA was significantly increased in the cortex and cerebellum in response to aging or after WNV infection. Notably, WNV infection also induced *Lgals3bp* mRNA in the hippocampus. *P2ry12* mRNA remained stable in response to these stimuli (Figure 1C).

### 3.2. Spatial Imaging Reveals Infection and Aging-Dependent Transcriptomic Changes in Mouse Brain

**Figure 2:**
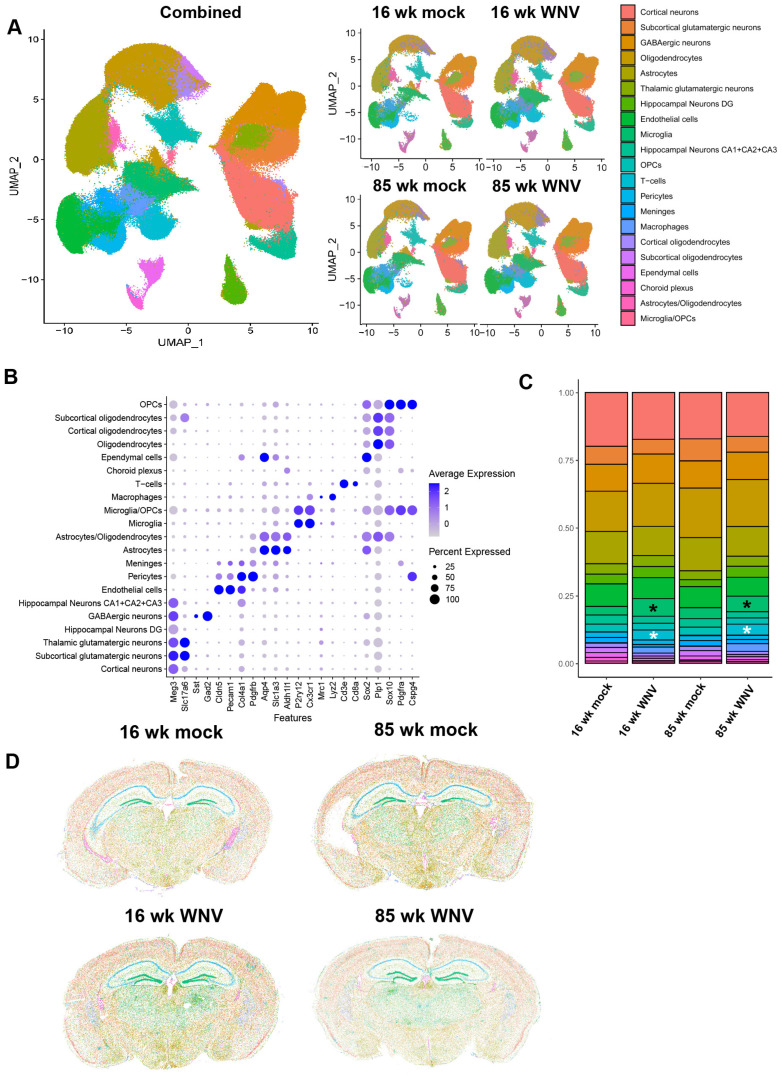
MERFISH analysis of WNV- and mock-infected mouse brains. (**A**) UMAP scatter plot showing clustering of identified cell types, combined, or split per sample type. (**B**) Dotplot of expression levels for gene markers used to identify cell types. (**C**) Frequency plot of identified cell types in each group; black and white asterisks denote microglial and T-cell populations, respectively. (**D**). Representative sections from mouse brains demonstrating identified cell types in 16- and 85-week-old mouse brains 30 days post infection with WNV or mock control. Color legend is consistent with (**A**).

To deeply profile infection- and age-dependent changes in mouse brain, we used spatial transcriptomics and a custom-designed panel consisting of 426 mouse genes involved in inflammatory response, aging, and senescence, in conjunction with established markers for identifying specific cell types in the mouse CNS. Specifically, the panel included 50 senescent markers of the SenMayo gene set [31], as well as a number of interferon-stimulated genes (ISGs) such as *Ifi204*, *Ifitm2*, *Ifitm3*, *Ifit1* and others. Analyses of forebrains obtained from WNV- or mock-infected, 16- or 85-week-old animals isolated at 30 DPI identified 20 cell clusters in coronal sections (Figure 2A) using a custom panel of markers for each cell type (Figure 2B). Identified frequency of each cell type (Figure 2C) and UMAP clustering analysis (Figure 2D) revealed expansion of microglia and oligodendrocytes in aged mice, as previously reported [32,33]. As previously reported in younger animals [7], WNV infection resulted in expanded microglial populations (Figure 2C, black asterisks) and CD8^+^ T cell infiltration (Figure 2C, white asterisks). We proceeded to use SenMayo gene module score to identify senescent cells in all four groups of samples (Supplementary Figure S1). This analysis revealed an increase in the fractions of senescent microglia and astrocytes in aged and infected samples, with aged infected samples having the highest fraction of senescent cells. Overall, these data suggest that a neuroinflammatory stimulus can promote accelerated acquisition of senescent phenotypes in glial cells, with astrocytes, microglia, and oligodendrocyte precursor cells (OPCs) being most affected.

### 3.3. Lgals3bp mRNA Is Broadly Expressed in the CNS by Microglia, Astrocytes, Neurons and, Most Prominently, by Ependymal Cells and the Choroid Plexus

**Figure 3:**
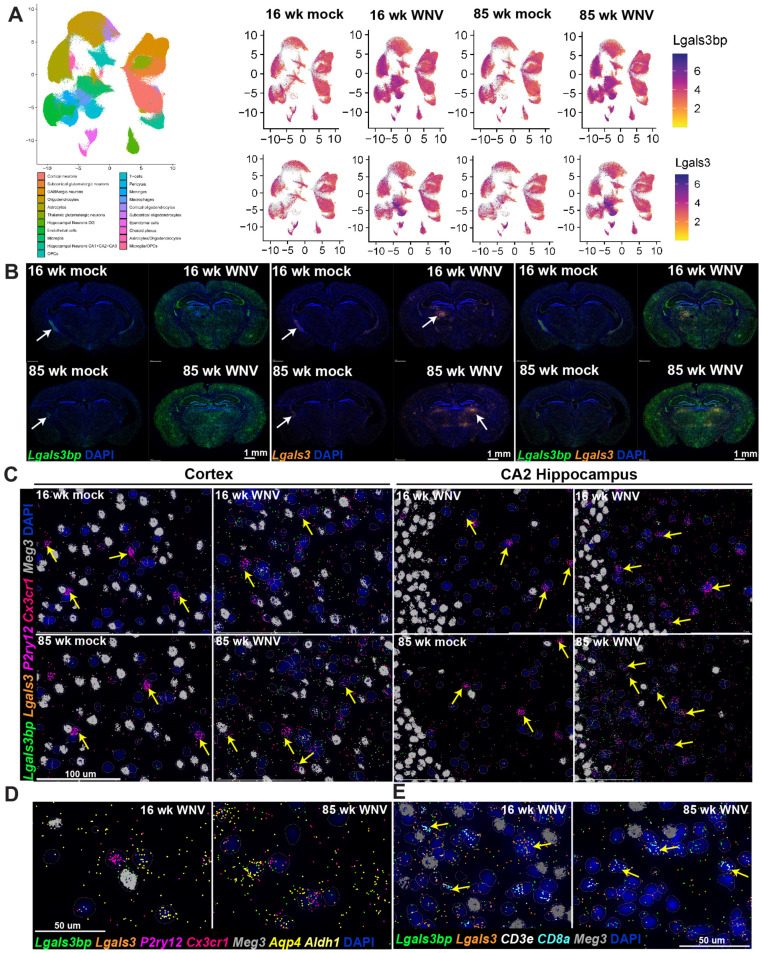
*Lgals3bp* expression in mouse brain visualized using MERFISH. (**A**) *Lgals3bp* and *Lgals3* expression scatter plot in identified cell types. (**B**) Spatial imaging of *Lgals3bp* (green) and *Lgals3bp* (orange) in coronal section of mouse brains from 16- or 85-week-old animals infected with mock or WNV, visualized at 30 DPI. Arrowheads point at persistent inflammatory foci observed in infected samples. Scale bar = 1 mm. (**C**) *Lgals3bp* (green), *Lgals3* (orange), *P2ry12* (magenta), *Cxcr1* (coral), and *Meg3* (gray) expression in mouse cortex (left panel) or CA2 region of the hippocampus (right panel). Arrowheads indicate microglial cells. Scale bar = 100 µm. (**D**) *Lgals3bp* (green), *Lgals3* (orange), *P2ry12* (magenta), *Cx3cr1* (coral), *Meg3* (gray), *Aqp4* (yellow), and *Aldh1* (light yellow) expression in the CA2 region of the hippocampus of WNV-infected mice. Scale bar = 50 µm. (**E**) *Lgals3bp* (green), *Lgals3* (orange), *CD3e* (white), *CD8a* (cyan) and *Meg3* (gray) expression visualized in inflammatory foci observed in the brain of WNV-infected animals at 30 DPI. Arrowheads point at CD8^+^ cells. Scale bar = 50 µm. DAPI staining is in blue across the figure.

Using spatial imaging data, we next established the pattern of *Lgals3bp* expression in brains of 16- and 85-week-old mock- or WNV-infected animals. UMAP scatterplots (Figure 3A) combined with visualization of spatial imaging data (Figure 3B) demonstrated that *Lgals3bp* mRNA is expressed in the CNS by microglia, astrocytes, and neurons. The highest expression levels of *Lgals3bp* in the absence of WNV infection were found in the ependymal cells and the choroid plexus (white arrows, Figure 3B, left panel). At 30 days post WNV infection, *Lgals3bp* expression was significantly increased throughout the brain, with cortex, hippocampus (specifically the CA2 region), and the subcortical white matter all expressing higher levels than uninfected controls (Figure 3B, left panel). Steady-state expression of *Lgals3* (Galectin 3) mRNA, to which LGALS3BP is a binding partner, was considerably more restricted, with ependymal cells and the choroid plexus expressing the highest amounts (Figure 3C, middle panel). WNV infection also resulted in increased *Lgals3* expression, but compared to *Lgals3bp*, the pattern of expression was less diffuse and more concentrated near local foci of inflammation (Figure 3B, middle panel, red arrows). Homeostatic microglia, identified by markers *Cx3cr1* and *P2ry12*, in the cerebral cortex (Figure 3C, left panel) expressed a limited but detectable amount of *Lgals3bp*. WNV infection resulted in a visibly higher fraction of *Lgals3bp*-expressing microglia (Figure 3C, yellow arrowheads), especially in aged 85-week-old animals. Similar findings were observed in the CA2 region of the hippocampus of mock-infected and WNV-infected animals. Neurons, identified by expression of *Meg3* mRNA, also expressed *Lgals3bp* and *Lgals3* (Figure 3D). *Aldh1^+^Aqp4^+^* astrocytes were also notable for relatively higher levels of *Lgals3bp* expression, especially in WNV-infected samples, and were often found in close proximity to neurons or *Lgals3bp*-expressing microglia. Upon closer examination of persistent inflammatory foci, notable for a significant increase in *Lgals3* expression (see Figure 3B, middle panel), we detected numerous infiltrating CD8 T cells, identified by expression of CD8a and CD3e markers (Figure 3E). These infiltrating T cells often expressed both *Lgals3bp* and *Lgals3*, and were proximal to neurons, which also expressed both of these molecules. Overall, these findings establish a pattern of *Lgals3bp* expression in the CNS at baseline, as well as after WNV infection, and indicate roles for Lgals3bp in recovery from WNV encephalitis.

### 3.4. WNV Infection of Lgals3bp^−/−^ C57BL6/J Mice Exhibits Increased Severity of Infection without Differences in Virologic Control or Survival

**Figure 4:**
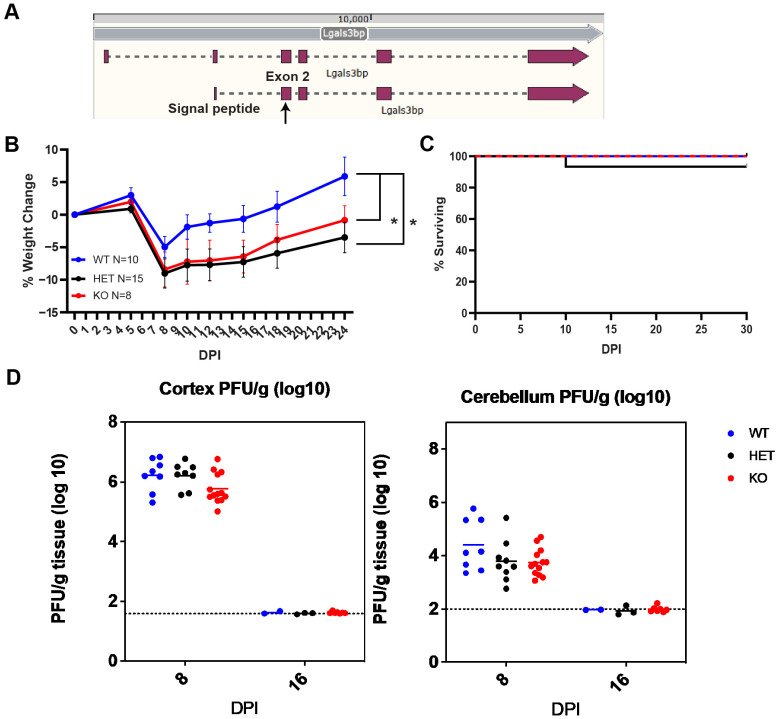
*Lgals3bp* KO C57BL6/J mice infected with WNV-NS5-E218A suffer increased severity of infection without a difference in survival or CNS viral titers. (**A**) Schematic demonstrating targeting sites of gRNA in exon 2 (arrow) of C57BL6/J mouse *Lgals3bp* gene used to generate *Lgals3bp^−/−^* animals. Weight loss (**B**) and survival (**C**) of WNV-infected *Lgals3bp^−/−^*, *Lgals3bp^+/−^* and WT animals. (**D**) CNS viral titers of WNV measured in the cortex and cerebellum via plaque assay. * denotes a *p*-value less than 0.05.

To determine whether *Lgals3bp* impacts acute infection with WNV, we generated *Lgals3bp^−/−^* on the C57BL6/J background by targeting exon 2 of the *Lgals3bp* gene using CRISPR-Cas9 to induce a frameshifting mutation (Figure 4A). Selected founder animals were backcrossed to wild-type C57BL6/J animals to address any potential mosaicism, with each generation genotyped via next-generation sequencing to identify homozygous mutations in the *Lgals3bp* gene. In addition, qRT-PCR of RNA obtained from brains of these mice confirmed more than a 90% decrease in *Lgals3bp* mRNA (Supplemental Figure S2). These *Lgals3bp^−/−^*, *Lgals3bp^+/−^* and wild-type (WT) C57BL6/J mice were infected with WNV at 8 weeks old and evaluated for survival and weight loss. This analysis revealed that, although *Lgals3bp^−/−^* and *Lgals3bp^+/−^* mice lost significantly more weight compared to their WT littermates (Figure 3B), there were no differences in survival, CNS viral titers, and time to clearance between the genotypes (Figure 3C,D).

### 3.5. Lgals3bp^−/−^ C57BL6/J Mice Exhibit More Microglia and CD4^+^ T Cells during WNV Infection

**Figure 5:**
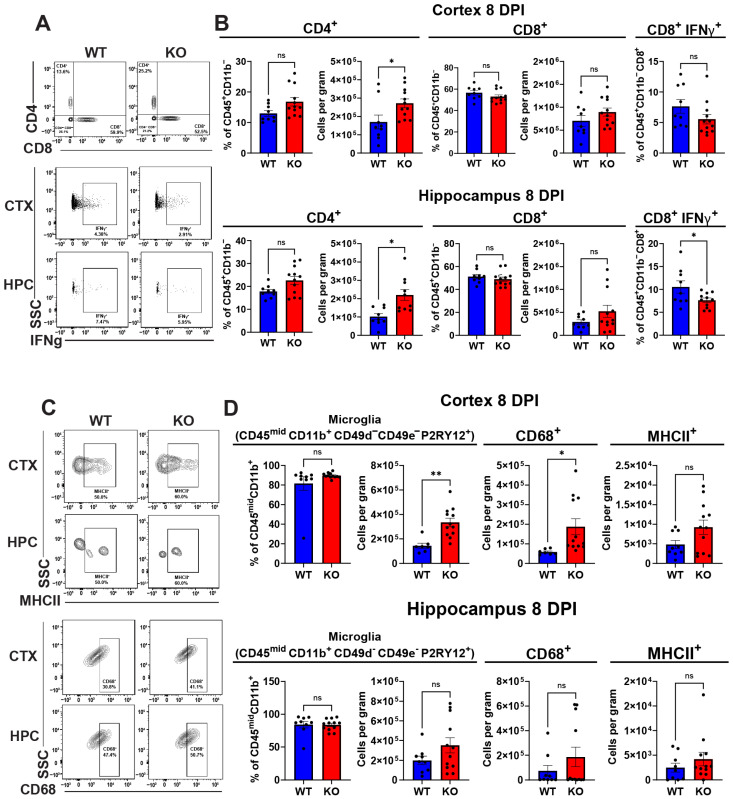
Flow cytometric analysis of lymphoid cells and microglia isolated from WNV-infected *Lgals3bp*^−/−^ and WT animals at 8 DPI. (**A**,**B**) Proportions and cell counts of CD4^+^ and CD8^+^ T cells isolated from cortices and hippocampi of *Lgals3bp*^−/−^ and WT mice at 8 DPI. Fraction of cells positive for IFNγ expression is shown in the right panel. (**C**,**D**) Proportion and cell count of microglia isolated from cortices and hippocampi of *Lgals3bp*^−/−^ and WT mice at 8 DPI. Counts of microglia expressing CD68 or MHCII are shown in the lower panel. * and ** denote *p*-values less than 0.05 and 0.005, respectively. ns denotes non-significant differences.

Because severity of illness during viral encephalitis can be due to the extent of CNS inflammation, we analyzed mononuclear cell infiltration and microglial phenotype within the forebrains of WNV-infected 8-week-old *Lgals3bp*^−/−^ versus WT mice at 8 DPI. Analysis of lymphoid cells (Figure 5A,B) revealed a significant increase in total number, but not in the fraction of CD4^+^ T cells in both cortex and hippocampus of *Lgals3bp*^−/−^ mice compared to similarly infected WT littermates. We also detected a significant reduction of IFNγ^+^CD8^+^ T cells in the hippocampi of *Lgals3bp^−/−^* mice compared with WT animals. A similar trend was observed in the cortex, but the results were not statistically significant. Overall numbers of CD8 T cells were not significantly reduced, and neither were numbers of TNF^+^ or WNV-specific T cells, or numbers of natural killer cells (Supplementary Figure S3). Significant increases in CD45^mid^CD11b^+^ CD49d^−^ CD49e^−^P2ry12^+^ microglia were detected within the cerebral cortices, but not the hippocampi, of *Lgals3bp*^−/−^ mice compared with WT animals (Figure 5C,D). These microglia were also notable for higher expression of CD68, a marker of microglia phagocytic activity, but not MHC-II. Overall, these data indicate that *Lgals3bp* deficiency results in increased numbers of microglia and CD4^+^ T cells during WNV infection.

## 4. Discussion

Aging is a complex biological process often accompanied by chronic low-grade inflammation, a phenomenon that has been referred to as “inflammaging” [34]. While most of the existing research aims at trying to better understand the inflammatory phenotypes that occur with aging, little is known about the capacity of an inflammatory stimulus to induce, or accelerate, acquisition of an aged or senescent phenotype. Some recent studies, however, have made significant progress: for example, Breen et al. examined epigenetic changes during HIV infection, and found that HIV infection leads to an approximately 2–5 years’ worth of accelerated senescence measured by alterations in the epigenome [35].

Accelerated epigenetic aging was also reported for those living in areas where leprosy is endemic [36] and, more recently, in patients after SARS-CoV-2 infection [37]. In this study, we used spatial imaging, flow cytometry and RNA sequencing to evaluate neuroinflammatory changes that occur following infection with WNV and to compare them to those that occur with aging. We focused on LGALS3BP, a potential novel marker at the intersection of senescence and inflammation and found that *Lgals3bp* levels are elevated during aging and WNV-induced neuroinflammation. We then genetically ablated *Lgals3bp* in mice, which led to an increase in the number of homeostatic microglia and CD4^+^ T cells in the brain combined with a decrease in IFNγ+ CD8^+^ T cells in the hippocampus, an area of the brain important for memory and neurogenesis and particularly affected by WNV infection [27,38].

We began by comparing the dataset generated by single-cell RNA sequencing of WNV-activated microglia [12] to a published dataset of RNA sequencing performed on aged microglia [28], which revealed broad similarities between WNV- and aging-induced transcriptomic changes in microglia: genes induced by microglial activation in the context of WNV infection were also induced by aging, while homeostatic microglial genes maintained consistent expression levels between the two conditions. Microglial activation has been previously reported during aging and recovery from WNV in mice [1,11,12,39,40,41], with a number of inflammation-induced genes being implicated in both of these conditions.

Next, we used spatial transcriptomics to profile WNV- and aging-induced changes in the mouse brain using a custom panel of 426 genes that included markers for CNS cell types, inflammatory molecules, and senescence genes, the latter with a particular focus on genes included in the SenMayo dataset [22]. This technology has recently been used to analyze changes in the mouse brain during aging [42], and it has been observed that during the course of aging, non-neuronal cell types, especially astrocytes and microglia, undergo more-pronounced changes in gene expression and spatial organization compared to neurons. When mice in this study were systemically administered lipopolysaccharide injection (LPS) to induce a severe inflammatory response, the authors noted both similarities and differences in cell-activation patterns produced by inflammation and aging. We also observed expansion of microglia and astrocytes with aging, and computing a SenMayo gene score for all cell types revealed an increase in senescent astrocytes and microglia, with mock uninfected samples having the fewest senescent cells, while aged infected samples had the highest fraction of senescent cells. These results, if confirmed by further studies, show that a neuroinflammatory stimulus can promote accelerated senescence in certain CNS cell types. Care needs to be taken, however, when interpreting these data, as the number of samples was low, and whether this aged phenotype is sustained long-term is unknown.

We also utilized spatial imaging to analyze the expression of *Lgals3bp* in mouse brain, which revealed a broad pattern of expression, especially when compared to a more restricted expression of *Lgals3. Lgals3bp* expression was shown to be increased in an aging-dependent manner in a published dataset of aged microglia [28], and we confirmed this finding using qRT-PCR of whole brains and bulk RNA sequencing of CD11b+ cells obtained from mouse brains. MERFISH imaging, however, did not demonstrate a robust increase in *Lgals3bp* with aging in uninfected mice, which can be attributed to an inherently less quantitative nature of this method, as well as a low number of samples in this analysis, leading to a decrease in statistical power. These data suggest that *Lgals3bp* expression is only moderately increased in an aged uninfected animal, but this difference becomes more apparent after WNV recovery when comparing infected 16- and 85-week-old mice (see heatmap in Figure 1B).

To address the function of *Lgals3bp*, we genetically ablated *Lgals3bp* in the C57BL6/J mouse strain. Another mouse with global gene deletion of *Lgals3bp* had been previously generated [43,44], but was unavailable. The previously published *Lgals3bp*^−/−^ was generated by targeting exon 3 of the *Lgals3bp* gene in a C57BL6/N mouse, while our mouse was produced by targeting exon 2 and was on the C57BL6/J background. C57BL6/J and C57BL6/N sub-strains are quite similar, but not identical, so care needs to be taken when comparing results obtained using different murine sub-strains. We chose the C57BL6/J to be consistent when comparing any new data to data obtained in our laboratory using our WNV infection and recovery model. WNV infection resulted in higher weight loss in *Lgals3bp*^−/−^ and *Lgals3bp*^−/+^ animals compared to wild-type littermates, but no increase in lethality or CNS viral titers. Previously generated *Lgals3bp*^−/−^ animals were noted for increased susceptibility to infections [16], however, an attenuated WNV strain was used in our study, which can potentially explain the lack of increased lethality or higher viral titers. This can potentially be addressed in future experiments by using another WNV strain, such as New York 1999, which we have previously used to show that aged mice are more susceptible to WNV infection [23].

Further analysis of neuroinflammation in WNV-infected *Lgals3bp^−/−^* mice revealed higher numbers of CD4^+^ T cells in both cortex and hippocampus compared with wild-type animals. However, a significant reduction of IFNγ^+^CD8^+^ T cells in the hippocampi of *Lgals3bp^−/−^* mice compared with WT animals was observed, as was an increase in numbers of homeostatic microglia, which reached significance in the cortex. We and other researchers previously demonstrated that IFNγ^+^CD8^+^ T cells that persist in the forebrain after recovery from RNA viral infections maintain microglial activation, synapse elimination and impairments in spatial learning and memory [13]. Thus, the changes in numbers and phenotypes of microglia, as well as CD4^+^ T cells in the context of *Lgals3bp* deletion suggest a novel possible target for targeting microglial-dependent post-infectious cognitive effects that are similar to those induced by aging. Future studies examining neural correlates of learning and memory are needed to determine whether *Lgals3bp* decelerates microglial activation phenotypes and their consequences during aging or after viral infections, as well as a deeper analysis of changes to microglial phenotype and the potential mechanisms of *Lgals3bp* action.

## 5. Limitations of the Study

This study looked at the effects of aging and West Nile Virus infection on neuroinflammatory processes in the brain and focused on the role of *Lgals3bp* during aging and infection. The work presented here has several limitations that should be considered when interpreting the results. First, the use of a single strain of West Nile Virus (WNV-NS5-E218A) may limit the generalizability of the findings to other strains of WNV or potentially other neurotropic infections. Furthermore, the accelerated acquisition of senescent phenotypes by the cells of the CNS most likely is not specific to WNV, as is a response to an inflammatory stimulus that can occur regardless of the origin. Additionally, this study primarily focused on male mice, which overlooks potential sex-specific differences in neuroimmune responses and microglial activation. Previous research indicates that male and female mice can exhibit distinct immune profiles and responses to neuroinflammation [45,46]. Finally, the scope of the study was limited to 30 days post-infection, which may not capture longer-term effects and the full extent of microglial senescence and neuroinflammation. Longitudinal studies are needed to determine the persistence of these phenotypes and their implications for long-term cognitive function and neurodegeneration. These limitations highlight the need for further research to validate and extend our findings, ideally incorporating diverse inflammatory stimuli, mouse models, sexes, and longer observation periods.

## Figures and Tables

**Table 1:** Transcriptional changes of cluster-defining genes of WNV-activated microglia during aging.

Gene	Fold Change (Old/Young) [28]	*p* Value [28]	Fold Change (WNV/Mock) [12]	*p* Value [12]
*Apoe*	3.56	5.8 × 10^−18^	4.46	2.93 × 10^−38^
*H2-D1*	3.29	1.8 × 10^−20^	8.29	1.46 × 10^−170^
*Fth1*	2.74	3.0 × 10^−18^	1.41	8.71 × 10^−11^
*Lgals3bp*	2.24	5.7 × 10^−23^	5.65	2.20 × 10^−121^
*H2-Q6*	2.23	1.1 × 10^−10^	2.29	0.00
*Oasl2*	1.89	1.9 × 10^−5^	15.52	1.08 × 10^−85^
*Ifi204*	1.79	1.0 × 10^−4^	12.84	4.66 × 10^−76^
*Fcgr4*	1.75	2.6 × 10^−4^	7.22	7.98 × 10^−53^
*C1qa*	1.75	4.5 × 10^−6^	−1.27	1.73 × 10^−46^
*Ifitm3*	1.72	4.0 × 10^−13^	10.02	8.85 × 10^−232^
*ltm2b*	1.63	6.4 × 10^−5^	−1.06	0.01
*Ftl1*	1.51	0.022	1.28	0.759
*Tyrobp*	1.50	0.002	−1.40	3.63 × 10^−44^
*Ctsb*	1.47	1.8 × 10^−4^	1.15	1.27 × 10^−9^
*Rps29*	1.39	0.212	1.68	1.92 × 10^−17^
*C1qb*	1.37	0.026	1.01	0.327
*Fau*	1.36	0.213	2.30	4.64 × 10^−48^
*Fcer1g*	1.35	0.010	−1.14	1.36 × 10^−37^
*H2-Ab1*	1.34	0.002	9.06	2.17 × 10^−166^
*Ctss*	1.32	0.002	1.23	2.40 × 10^−8^
*Ly6e*	1.26	0.005	1.97	3.54 × 10^−24^
*H2-Eb1*	1.25	0.014	7.52	3.96 × 10^−176^
*H2-Aa*	1.15	0.326	8.92	5.82 × 10^−180^
*CD74*	1.14	0.404	11.45	3.51 × 10^−213^
*Cxcl9*	1.12	0.722	261.85	2.15 × 10^−27^
*Psap*	0.93	0.846	−1.03	6.49 × 10^−23^
*Selplg*	1.11	0.652	−2.50	2.40 × 10^−25^
*Fscn1*	1.04	0.917	-5.40	8.67 × 10^−207^
*Hexb*	1.00	0.986	−2.41	3.23 × 10^−606^
*Cst3*	0.99	0.964	−3.03	1.52 × 10^−127^
*Hpgd*	0.88	0.326	−2.57	1.65 × 10^−80^
*Pmp22*	0.86	0.149	−5.41	2.97 × 10^−166^
*Selenop*	0.85	0.242	−2.60	5.19 × 10^−87^
*Csf1r*	0.84	0.241	−2.58	5.73 × 10^−88^
*Sparc*	0.82	0.237	−2.96	5.21 × 10^−11^
*Rnase4*	0.80	0.007	−2.78	2.80 × 10^−85^
*Golm1*	0.74	0.091	−3.32	1.35 × 10^−98^
*Siglech*	0.73	0.002	−2.85	3.28 × 10^−112^
*Cx3cr1*	0.73	0.157	−3.07	3.42 × 10^−99^

## Data Availability

Data are contained within the article and supplementary materials, RNA-seq data for Figure 1 is accessible via Gene Expression Omnibus (GEO), accession number GSE270687.

## Supplementary Materials

The following supporting information can be downloaded at: https://www.mdpi.com/article/10.3390/biom14070808/s1, Figure S1: MERFISH analysis of senescent cells from WNV- and mock-infected mouse brains. Figure S2: qRT-PCR analysis of *Lgals3bp* expression in wild type C57BL6/J and Lgals3bp KO mouse. Figure S3: Flow cytometric analysis of NK cells, NS4+ WNV-specific T cells and TNFa+ CD8 cells from WNV-infected *Lgals3bp*^−/−^, and WT animals at 8 DPI.
