# Supplementary material for: West Nile Virus-Induced Expression of Senescent Gene Lgals3bp Regulates Microglial Phenotype within Cerebral Cortex
Source: Biomolecules. 2024 Jul 8;14(7):808. doi: 10.3390/biom14070808 (PMC11274721; doi:10.3390/biom14070808)
Supplement: Supplementary file 1 [file biomolecules-14-00808-s001.zip › biomolecules-3058206-supplementary.pdf]

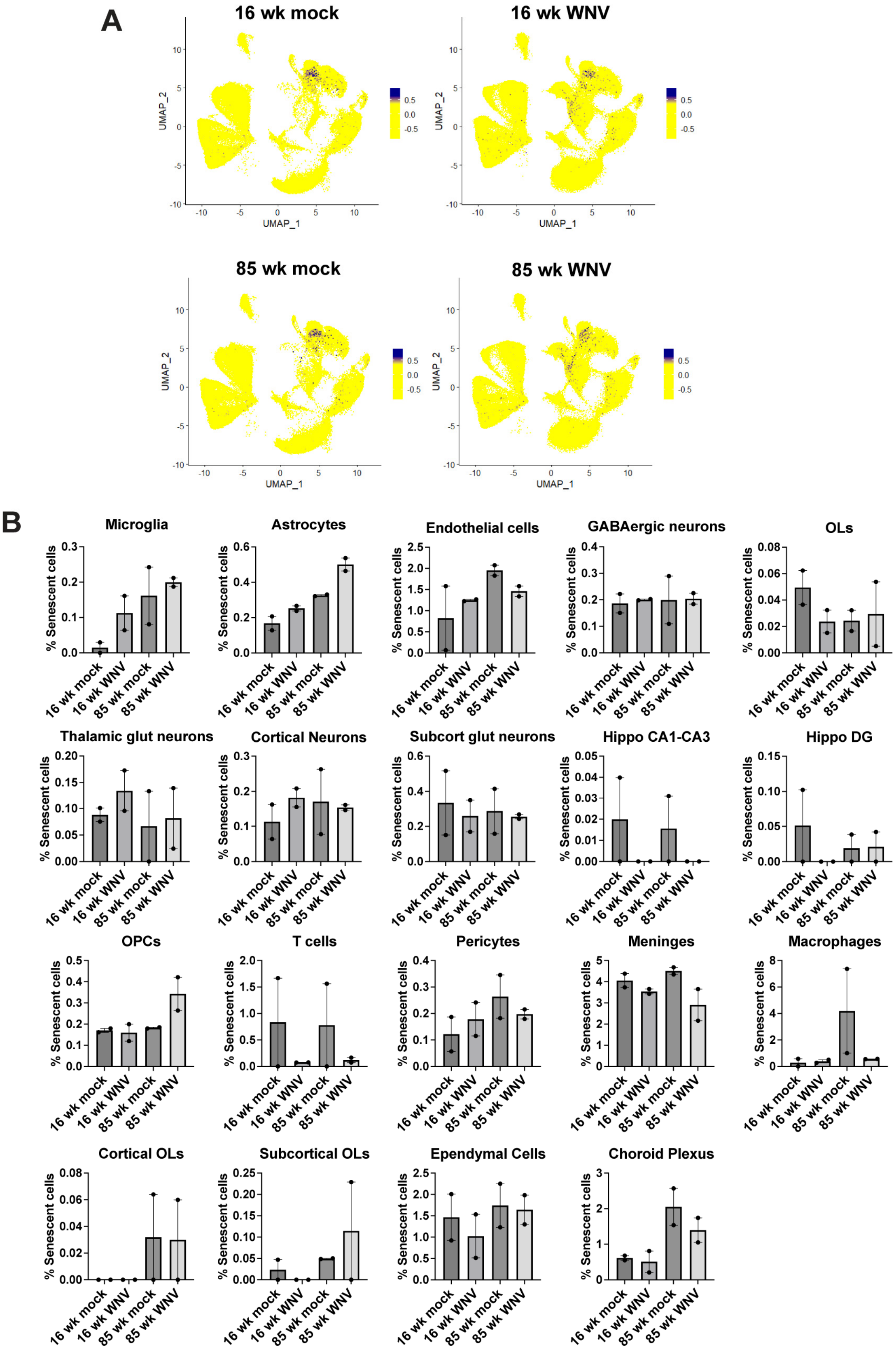

Figure S1. MERFISH analysis of senescent cells from WNV- and mock-infected mouse brains. (A) Scatterplots of senescent cells (dark blue) identified by a SenMayo gene module score per sample type. (B) Bar chart showing percentages of senescent cells of each of identified cell type, split by sample type.

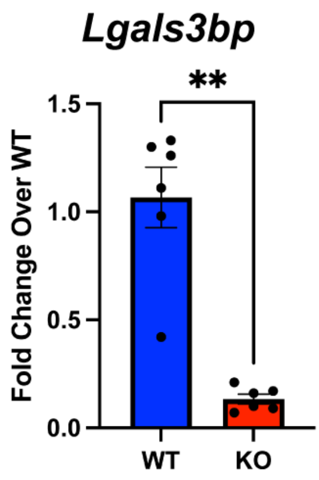

Figure S2. qRT-PCR analysis of *Lgals3bp* expression in wild type C57BL6/J and Lgals3bp KO mouse.

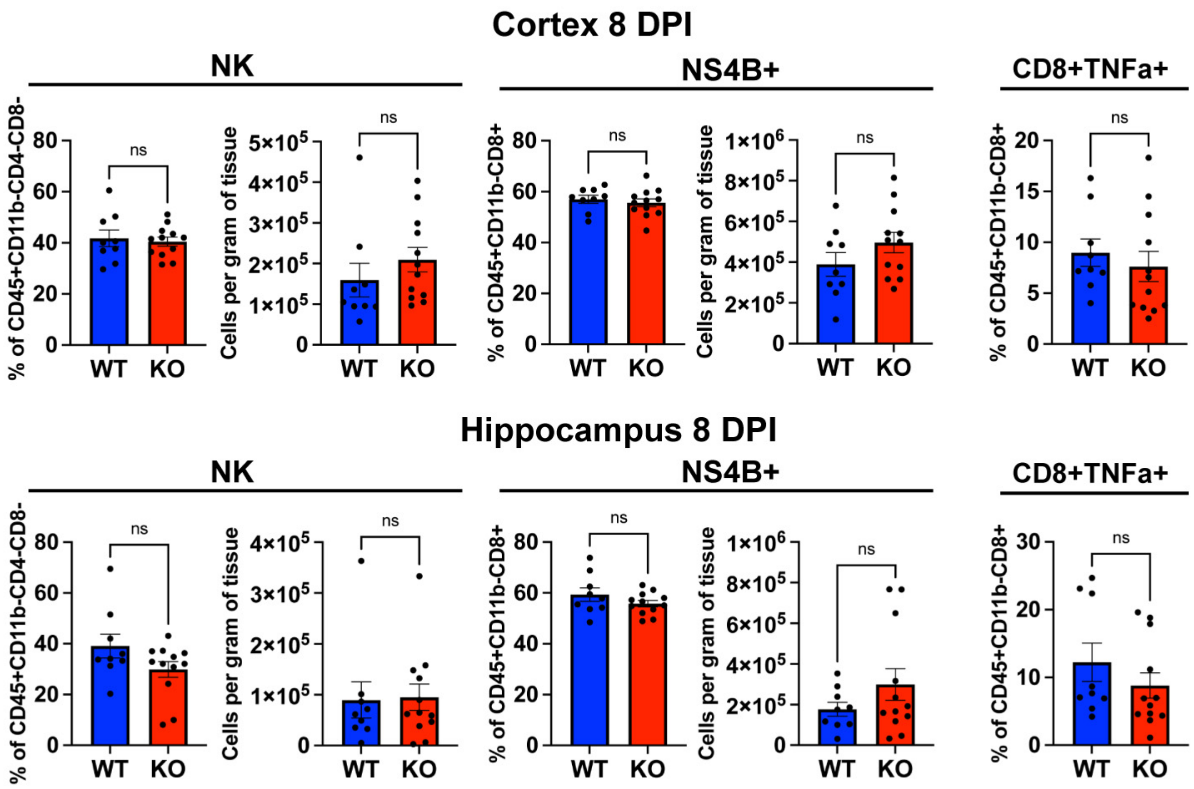

Figure S3. Flow cytometric analysis of NK cells, NS4+ WNV-specific T cells and TNFa+ CD8 cells from WNV-infected *Lgals3bp-/-*, and WT animals at 8 DPI.
